# Supplementary figures and images for: PIK3R1W624R Is an Actionable Mutation in High Grade Serous Ovarian Carcinoma
Source: Cells. 2020 Feb 14;9(2):442. doi: 10.3390/cells9020442 (PMC7072782; doi:10.3390/cells9020442)

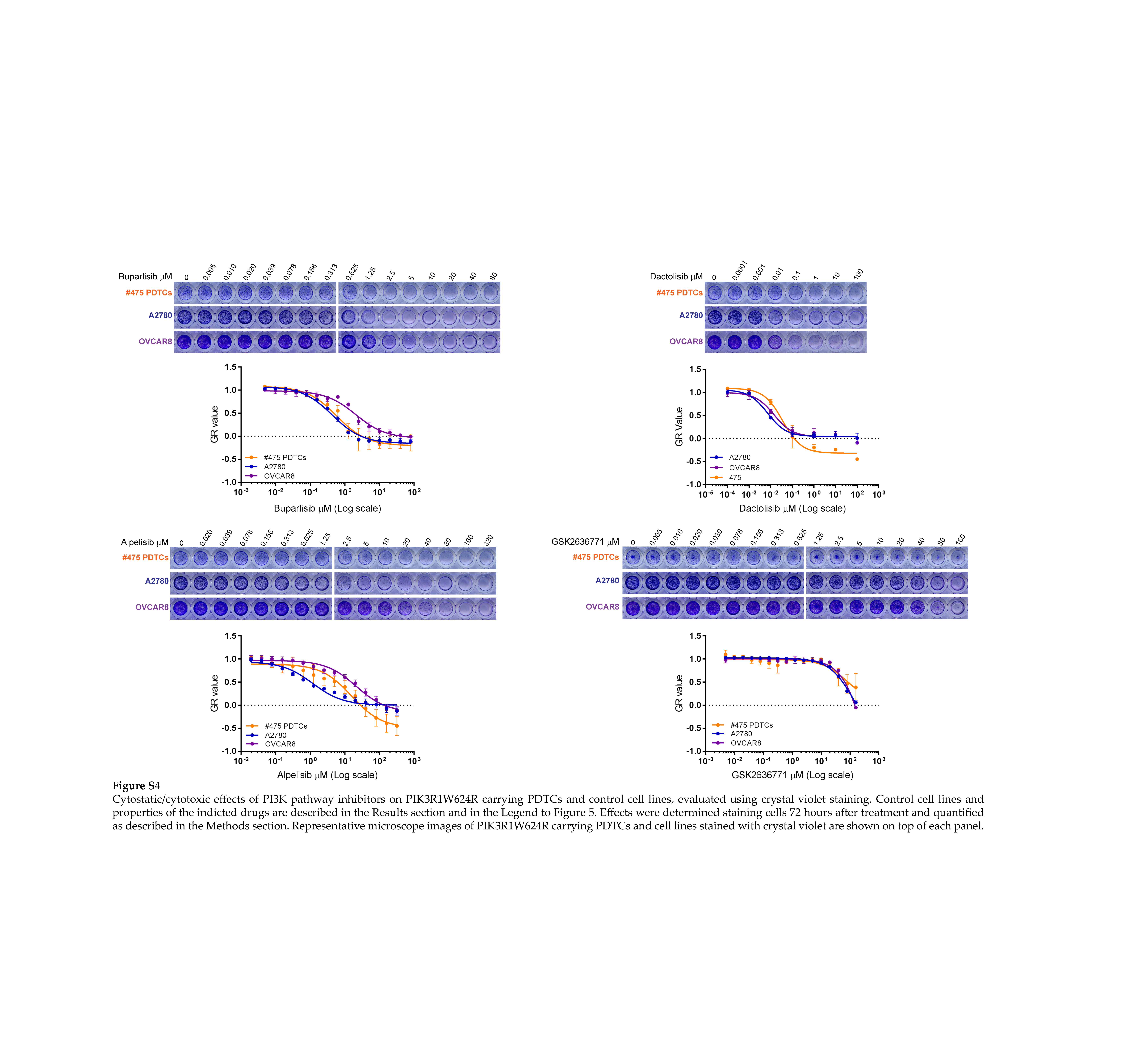

Supplement: Supplementary file 1 [file cells-09-00442-s001.zip › cells-650255-supplementary material updated with respect to the uploaded /DAmbrosio_Fig S4.tif]

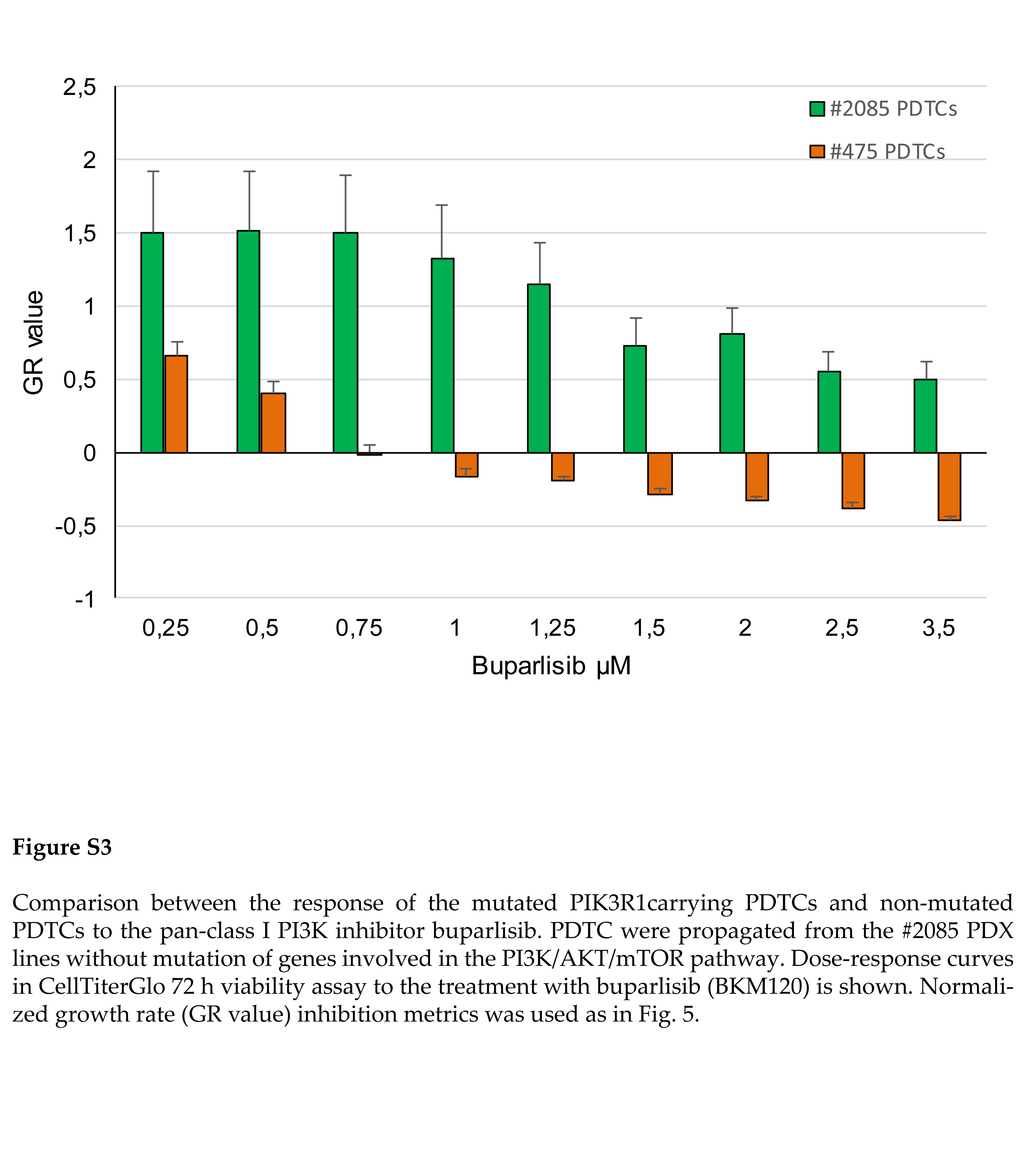

Supplement: Supplementary file 1 [file cells-09-00442-s001.zip › cells-650255-supplementary material updated with respect to the uploaded /DAmbrosio_Fig S3.tif]

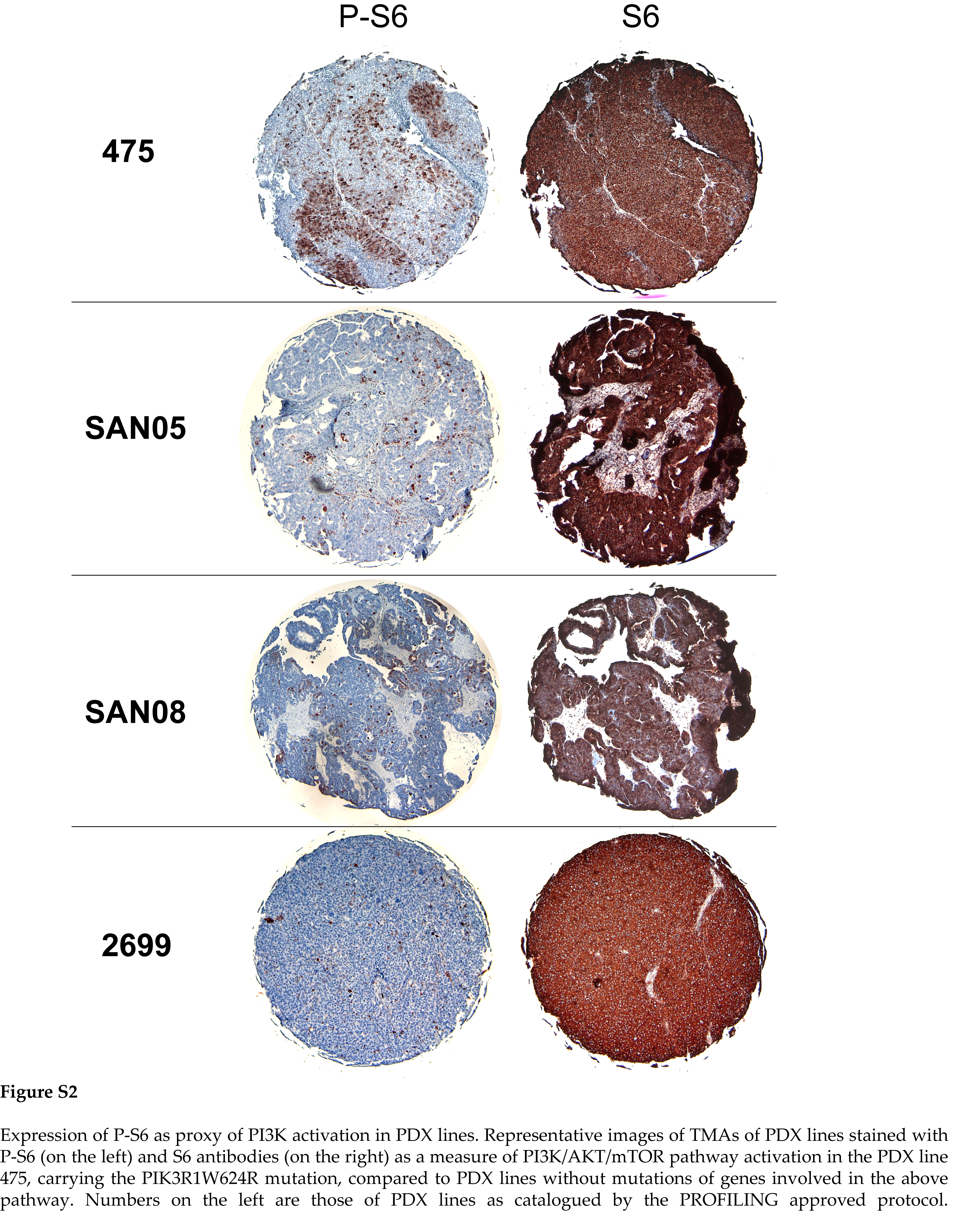

Supplement: Supplementary file 1 [file cells-09-00442-s001.zip › cells-650255-supplementary material updated with respect to the uploaded /DAmbrosio_Fig S2.tif]

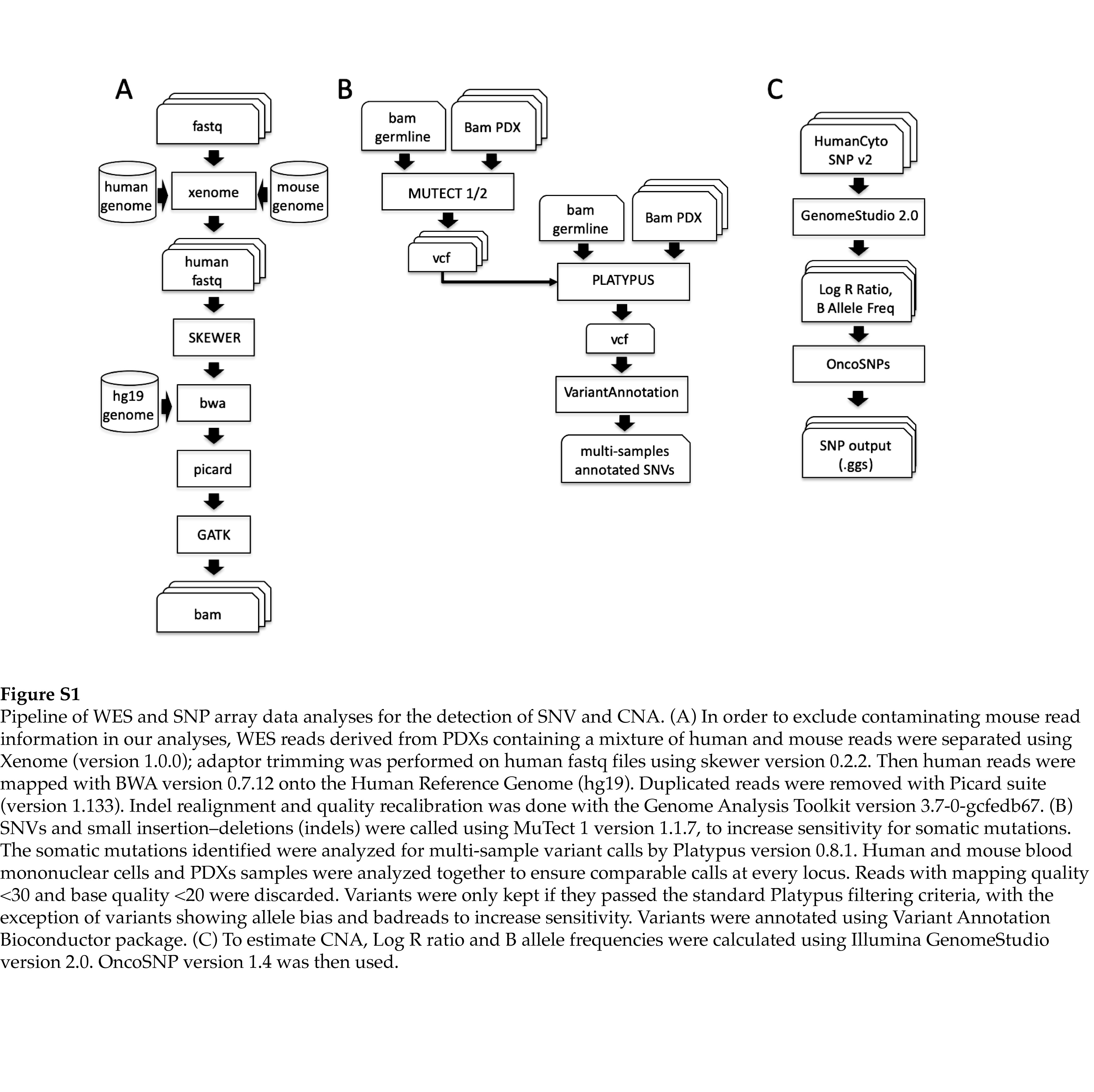

Supplement: Supplementary file 1 [file cells-09-00442-s001.zip › cells-650255-supplementary material updated with respect to the uploaded /DAmbrosio_Fig S1.tif]
