# Supplementary material for: PIK3R1W624R Is an Actionable Mutation in High Grade Serous Ovarian Carcinoma
Source: Cells. 2020 Feb 14;9(2):442. doi: 10.3390/cells9020442 (PMC7072782; doi:10.3390/cells9020442)
Supplement: Supplementary file 1 [file cells-09-00442-s001.zip › cells-650255-supplementary material updated with respect to the uploaded /DAmbrosio Suppl Methods Part 1.doc]

**Supplementary Methods Part 1**

*Patient Derived Xenograft (PDX) platform and animal treatment*

Only female NSG mice were used, obtained from Charles River Laboratories Italy. Used NSG mice did not receive any further modification. Mean weight at beginning of the experiments was approximately 22 gr. and slightly increased (up to 30 gr.) at the end of experiments. All mice belonging to a specific treatment group were kept in the same cage. Mice belonging to different treatment groups were never mixed. Refer to text for doses and treatment. All animals are monitored daily in the animal facility.

Mice were housed individually in cages with filtered ventilation (autoclaved polycarbonate cages), 5 to 7 female mice per cage, at 20 to 22 °C, on a 12:12-h light:dark cycle, and were given commercial pelleted diet. Autoclaved bedding material was highly adsorbing material without dust and was changed every week. Environmental enrichment was done using sterile red small cages.

Tumour fragments were grafted in a subcutaneous pocket (0.5 cm surgical cut) in anesthetized mice (gas anesthésia made of 2% isoflurane obtained from Zoetis U.K., in oxygen and nitrous oxide 2:1). Mouse skin was closed with surgical glue and stitches. Gas anesthésia was preferred because less irritant and immediately reversible upon discontinuation.

For *in vivo* treatment experiments, PDX tumour fragments from early passaged PDX and with comparable volumes were injected subcutaneously as above in 16-20 female mice. Mice to be utilized as either treated or control animal groups were blindly and individually randomized as described in the Methods section to be assigned to groups of 6-8 for each experimental group, and only subsequently identified. All groups were treated simultaneously for a given xenograft cohort.

For drug treatment, buparlisib (Cayman Chem. co., Ann Arbor, MI) was freshly diluted every day before treatment in N-Methyl-2-pyrrolidinone and PEG (both purchased from Sigma Aldrich (St. Louis, MO), 10/90 v/v to a final volume of 100 μl. The drug was administered by oral gavage every day at 10.00 a.m., 5 days/week, for 3 weeks, in sterile conditions in laminar flow hoods and using a small rigid cannula connected to a syringe. Buparlisib was administered at the dose of 20 mg/Kg. Control animals were treated with vehicle alone. Everything was carried in accordance with the protocol approved by the competent Italian authorities (see the Methods section). For example, anesthesia and analgesia for oral gavage carried out in these experiments were not administered because dangerous for this procedure. Animals were monitored immediately after treatment, then hourly and then daily for any inconvenience, which did not occur.

Mice in treatment were monitored for tumour take and growth twice a week. According to Italian Ministry of Health guidelines, mice were sacrificed under gas anaesthesia (see Methods section) by cervical dislocation when the tumour size reached 2000 mm^3^. All the outcomes and possible adverse event are hypothesised, discussed and subjected to approval of the local Ethical Commission and of the Italian Ministry of Health in accordance with **EU Directive 2010/63/EU for animal experimentation**. However, no euthanasia was necessary for deterioration of clinical conditions. All experiments were carried out taking into account the 3R principles.
